# Overcoming divalent cation sensitivity is not the only challenge for functional study of ABC transporters within polymer lipid particles

**DOI:** 10.1042/BSR20250256

**Published:** 2026-02-04

**Authors:** Olivia P. Hawkins, Thomas J. Potter, Luke M. Broadbent, Philip Kitchen, Alan D. Goddard, Alice J. Rothnie

**Affiliations:** Aston Institute for Membrane Excellence & Department of Biosciences, Aston University, Birmingham, U.K.

**Keywords:** AASTY, BmrA, DIBMA, MRP4/ABCC4, PLP, SMALP

## Abstract

Proteins of the ATP-binding cassette (ABC) transporter superfamily are involved in diverse biological processes including multidrug resistance. As membrane proteins, they exist within a complex lipid environment, and often it is necessary to isolate them from the other membrane components to study their structure, function, and dynamics. Traditionally, detergents have been used to isolate the transporters into micelles but this can strip away lipids that may be essential for function. Polymers such as styrene maleic acid (SMA) offer attractive alternatives to detergents as they retain the protein and lipids in a nanoscale disc. However, to date, no demonstration of full ABC transporter activity in these discs has been achieved, possibly due to the inherent divalent cation sensitivity of the SMA polymers; magnesium is essential for ATP binding to ABC transporters. Novel polymers such as those based on acrylic acid styrene (AASTY) show decreased sensitivity to divalent cations and, as such, may be well placed to probe ABC transporter activity. We have demonstrated that a range of commercially available AASTY polymers solubilise biological membranes efficiently, albeit with slightly different kinetics. ABC transporters can be solubilised and purified using AASTY polymers into discs of a comparable size to those formed by SMA2000. These discs show increased magnesium tolerance but, as for SMA2000, lipids within them do not seem to undergo a full phase transition. We were unable to detect ATPase activity of ABC transporters in AASTY polymer lipid particles, suggesting that magnesium tolerance alone is not sufficient to overcome the challenges.

## Introduction

Membrane proteins are critical for all life due to their activity in cellular defence, communication, and energy generation. They account for ~50% of current drug targets and understanding their structure and function is becoming increasingly essential for drug development [1,2]. One important group of membrane proteins are those belonging to the ATP-binding cassette (ABC) transporter superfamily. These transporters are present in all organisms from bacteria to man and utilise energy produced by ATP hydrolysis to power the transport of molecules across the cell membrane. The core structure of an ABC transporter comprises two nucleotide binding domains, where ATP binds and is hydrolysed, and two transmembrane domains where the transported substrates bind [3]. They can transport a chemically and structurally diverse range of substrates including xenobiotics and chemotherapy agents utilised in therapeutic pharmacology. Due to this activity, the mutation and/or overexpression of some ABC transporters have been shown to modify drug pharmacokinetics and lead to the development of multidrug resistance [4]. Other members of the family are involved in various genetic diseases [5]. For this study, we investigated two model ABC transporters—the bacterial *Bacillus subtilis* multidrug resistance ABC transporter (BmrA) [6] and mammalian multidrug resistance protein 4 (MRP4/ABCC4) [7].

Like many membrane proteins, ABC transporters can be difficult to work with due to their complexity and sensitivity once isolated from the plasma membrane. Traditionally, detergents have been utilised to solubilise and stabilise membrane proteins in detergent micelles. However, this method typically involves stripping away the native phospholipids and protein–lipid interactions that have been increasingly evidenced to affect protein structure and therefore function [8], thus there is a requirement for alternative methods of study.

Recent advancements in copolymer technology have established styrene maleic acid (SMA) as an efficient alternative solubilisation agent. Unlike detergents, copolymers can solubilise membrane proteins that remain embedded within their native phospholipid bilayer, forming SMA lipid particles (SMALPs) [9]. Both BmrA [10,11] and MRP4 [12,13] have been shown to be successfully solubilised using copolymers. However, the functional study of ABC transporters in copolymer nanodiscs remains challenging, with ATPase functionality not proven. The copolymer sensitivity to divalent cations causes precipitation of the target protein upon addition of magnesium, which is required as a cofactor for ATPase study [12,14]. Alternatively, it may be that tight lipid packing within SMALPs limits full conformational movement of the target protein, therefore preventing ATPase activity and functionality [15]. To probe these theories, alternative copolymers with increased magnesium tolerance were tested.

Acrylic acid styrene (AASTY) copolymers are commercially available at defined molecular weights (6 or 11 kDa) with varying percentages of acrylic acid moieties (45%, 50%, and 55%). They have been reported to be effective for membrane protein solubilisation and exhibit a decreased sensitivity to magnesium [16,17]. Diisobutylene maleic acid (DIBMA) has also been shown to have an increased tolerance to divalent cations [18,19], and so was also included in this study. The aim was to investigate whether overcoming Mg^2+^ sensitivity enabled measurement of ABC transporter ATPase activity within polymer lipid particles (PLPs).

## Methods

### Polymers

SMA2000 was provided as a kind gift from Cray Valley, supplied as SMAnh powder that required conversion into active SMA form. This was carried out as previously described [10,20]. DIBMA (monosodium salt) was purchased from Glycon Biochemicals and AASTY copolymers were purchased from Cube Biotech, all were supplied as a ready to use powder.

### Recombinant protein expression and membrane preparation

C41 (DE3) *Escherichia coli* were transformed with the vector pET23b-BmrA (kind gift from Prof. Jean-Michel Jault, IBCP, Lyon, France), cultured and harvested as described previously [10]. Briefly, 5 ml LB–amp (Luria Broth supplemented with 100 μg/ml ampicillin) was inoculated from a glycerol stock and grown overnight at 37°C. This was then used to inoculate a 1 l culture of LB–amp, which was grown at 37°C 200 rpm until the OD_600_ reached 0.6. Protein expression was induced by addition of 0.5 mM isopropyl β-d-1-thiogalactopyranoside, the temperature was decreased to 25°C and incubated overnight. Cells were then harvested by centrifugation (6000×***g***, 10 min).

MRP4 was expressed in Sf9 insect cells using a recombinant baculovirus system as described previously [12,21]. Briefly, Sf9 cells were grown in suspension culture at 27°C in ESF 921 insect cell culture medium supplemented with 10% foetal bovine serum, 100 U/ml penicillin, and 100 mg/ml streptomycin. Cells at 2 × 10^6^ cells/ml were infected with the recombinant baculovirus at an MOI of 2. After 48 h, cells were harvested by centrifugation (7000×***g***, 10 min, 4°C).

For both BmrA and MRP4, the cell pellets were resuspended in 20 ml buffer H (50 mM Tris, 0.25 mM CaCl_2_, and 250 mM Sucrose) including protease inhibitors (1 mM pepstatin, 1.3 mM benzamidine, and 1.8 mM leupeptin). Resuspended BmrA expressing *E. coli* cells were processed through a French Press (3 × 16,000 pounds per square inch), whereas MRP4 Sf9 cells were disrupted through nitrogen cavitation at 500 psi, 4°C. Cell lysates were centrifuged (750×***g***, 10 min, 4°C), the supernatant retained and ultracentrifuged (100,000×***g***, 20 min, 4°C). The membrane pellet was resuspended at a wet membrane weight of 60 mg/ml in buffer P (20 mM Tris and 150 mM NaCl, pH 8) and stored in aliquots at −80°C.

### Sf9 membrane solubilisation kinetics

Sf9 insect cell membranes (50 μl) at 60 mg/ml (wet pellet weight) were mixed rapidly with 50 μl 5% (w/v) SMA2000 or DIBMA, or 1% (w/v) AASTY polymers or buffer P, at room temperature in a cuvette with a 3 mm pathlength, and the light scattering was monitored over 5 min at 390 nm using a spectrophotometer (Pharmacia Biotech Ultrospec 2000). End point measurements were also taken after 1 h incubation. Experiments were repeated in triplicate for each polymer.

### ABC transporter solubilisation

Five percent (w/v) SMA2000 or DIBMA, or 1% (w/v) AASTY polymer or 2% (w/v) DDM in buffer P was added in equal volume to 60 mg/ml BmrA *E. coli* or MRP4 Sf9 membrane stocks and mixed for 1 h at room temperature. Samples were then ultracentrifuged (100,000×***g***, 20 min, 4°C) with the supernatant containing soluble membranes harvested. Pellets containing insoluble material were resuspended in the same volume of buffer P supplemented with 2% (w/v) sodium dodecyl sulfate (SDS). Samples of soluble and insoluble material were loaded on sodium dodecyl sulphate–polyacrylamide gel electrophoresis (SDS–PAGE) and transferred to PVDF membranes. Western blots were probed with either an anti-his primary antibody (R&D Systems MAB050, 1:5000) and an anti-mouse HRP conjugated secondary antibody (Cell Signalling), or M4_I_-10 anti-MRP4 primary antibody (Enzo ALX-801-038, 1:200) and an anti-rat HRP secondary antibody (Cell Signalling). They were developed with chemiluminescence (Pierce) and imaged with a C-Digit scanner (Licor). Solubilisation efficiency was calculated from densitometric analysis of the Western blots (ImageJ).

### Lipid extraction and thin-layer chromatography

BmrA *E. coli* or MRP4 Sf9 membranes were solubilised with selected copolymers, ultracentrifuged, and the soluble fraction harvested as described above. Lipids from the resulting PLPs, as well as control membrane samples were then extracted by methyl-tert-butyl ether (MTBE) method [22] whereby 1 ml samples were mixed with 300 μl MTBE: methanol (10:3) and incubated for 1 h on a shaker plate. Following incubation, 50 μl of dH_2_O was added to induce phase separation and samples were centrifuged (1000×***g***, 10 min, 4°C). The solvent layer was collected and then dried using nitrogen and stored at −20°C before thin-layer chromatography (TLC) analysis.

Lipid extracts were analysed by TLC using a chloroform:methanol:dH_2_O (65:25:4) solvent system with silica gel 60 F254 plates. Lipid extracts were solubilised using chloroform: methanol (1:1) and successively dotted onto the sample line alongside 25 μg of lipid controls (phosphatidylserine (PS), phosphatidylcholine (PC), phosphatidyl glycerol (PG), cardiolipin (CL), and phosphatidylethanolamine (PE)). The plate was stained with molybdenum blue. The relative abundance of key lipids in each sample were calculated using densitometry (Image J).

### Affinity purification of BmrA

Solubilised protein samples were mixed with washed HisPur Ni-NTA resin (100 μl of resin per ml of solubilised protein) and shaken gently overnight using a rotary mixer. The following day samples were transferred to a gravity column and washed five times with 10× bed volume (BV) of buffer P containing 20 mM imidazole. This was followed by two washes with 10× BV of buffer P containing 40 mM of imidazole. Elution fractions were then taken using 0.5× BV of buffer P containing 200 mM of imidazole solution. Samples of the different steps were analysed by SDS–PAGE and stained with Instant Blue (Abcam). For DDM solubilised protein the method was the same, but all wash buffers were supplemented with 0.1% (w/v) DDM.

To assess BmrA purity, a single lane of an SDS–PAGE gel showing purified BmrA was analysed by densitometry. To calculate BmrA yield, bovine serum albumin (BSA) standards (0.25, 0.5, 0.75, 1, 1.25, and 1.5 μg) were run on SDS–PAGE alongside purified BmrA and quantified using densitometry. Values obtained were corrected for membrane batch-to-batch variation in yield by normalising to the values obtained with SMA2000 using the same membrane preparation.

### Preparation of lipid only PLPs

1,2-Dimyristoyl-*sn*-glycero-3-phosphocholine (DMPC) lipids were dissolved in 2:1 chloroform:methanol and dried under nitrogen. The lipid film was resuspended in buffer P to form a 2% (w/v) suspension of DMPC liposomes. DMPC liposomes (2% (w/v)) were mixed with an equal volume of 1% (w/v) polymer at room temperature. To remove excess free polymer the sample was run on a Superdex 200 10/300 column (GE Healthcare), equilibrated with buffer P, at a flow rate of 0.25 ml/min, and absorbance detected at 280 nm. The fractions corresponding to the SMALP peak were collected and stored at 4°C.

### Magnesium sensitivity assay

Size exclusion chromatography (SEC)-purified lipid-only PLPs were mixed with various concentrations of MgCl_2_ (0–10 mM) in a 96-well plate, and light scattering was measured at 390 nm using a Multiskan Go plate reader [14].

### Dynamic light scattering

SEC-purified lipid-only PLPs were analysed by dynamic light scattering (DLS) using the Zetasizer Nano ZS (Malvern Panalytical) and polystyrene cuvettes (Sarstedt) with a path length of 1.0 cm, at a wavelength of 633 nm. One milliliter of concentrated SEC-purified lipid-only PLPs in buffer P was measured at 25°C with 120 s equilibration time. Each measurement was repeated at least 12 times and performed in triplicate with an average of the three runs obtained and analysed in Zetasizer Software 7.13 (Malvern Panalytical).

### Mass photometry

SEC-purified lipid-only PLPs were analysed using an MPTwo mass photometer (Refeyn). Samples were prediluted to approximately 100 nM in PBS, then diluted ten-fold into a PBS droplet on the photometer stage to give a final concentration of approximately 10 nM. Data were collected for 60 s, with the image size set to ‘regular’.

### Laurdan assay

To measure membrane fluidity, a laurdan assay was employed. Five microliters of 1 mM laurdan (in DMSO) was mixed with 500 μl of 0.1% (w/v) DMPC liposomes. This corresponds to a laurdan:lipid molar ratio of 1:150. Fluorescence of the laurdan was measured using a Perkin Elmer LS55 spectrophotometer, with an excitation wavelength of 350 nm and emission wavelength spectra of 380–600 nm, with 15 nm slit widths. The fluorescence spectra was read after incubating the lipid sample at temperatures from 4°C to 60°C. For assessment of the fluidity within PLPs the laurdan containing DMPC liposomes were mixed with 20 μl 5% (w/v) polymer for 30 min, then the fluorescence spectra measured at varying temperatures as above. The fluorescence intensity measurements (I) at 435 and 490 nm were utilised in analyses, with GP (general polarisation) value calculated according to the equation:

$$\text{GP}=\frac{\left({\text{I}}_{435}-{\text{I}}_{490}\right)}{\left({\text{I}}_{435}+{\text{I}}_{490}\right)}.$$

### Ligand binding assays

Substrate binding to purified BmrA was measured using a tryptophan fluorescence quenching assay as described previously [10]. Imidazole was removed from purified BmrA using centrifugal filter concentrators (Amicon Ultra, 30K cut-off or Pierce PES, 30K cut-off). Tryptophan fluorescence of BmrA (30 μg/ml) was monitored using a PerkinElmer LS55 fluorimeter, with an excitation wavelength of 280 nm (slit width 10 nm) and emission measured at 310–400 nm (slit width 20 nm). Fluorescence quenching by the successive addition (1–50 μM) of Hoechst 33342 was measured at 335 nm (λmax). Fluorescence intensities were corrected for the effects of dilution and the inner filter effect using N-acetyl tryptophanamide. Results were analysed by non-linear regression using GraphPad Prism to fit a one-site binding curve.

### ATPase assays

The ATPase activity of purified BmrA in PLPs or DDM micelles was measured using a colourimetric 96-well plate assay as previously described, which is based on the detection of inorganic phosphate released during ATP hydrolysis [23]. BmrA samples were incubated with varying concentrations of ATP (0–2 mM) in buffer A (50 mM Tris, 150 mM NH_4_Cl, 5 mM MgSO_4_) at 37°C for 20 min, then the reaction was stopped by addition of 5% (w/v) SDS. Colourimetric detection of Pi concentration was carried out by addition of ammonium molybdate and ascorbate as described previously [24]. Activity was calculated as nanomoles of Pi liberated per minute per milligram of pure protein, and data were fit by non-linear regression with a Michaelis–Menten curve (Graphpad Prism).

## Results

### Solubilisation of biological membranes using AASTY polymers

**Figure 1:**
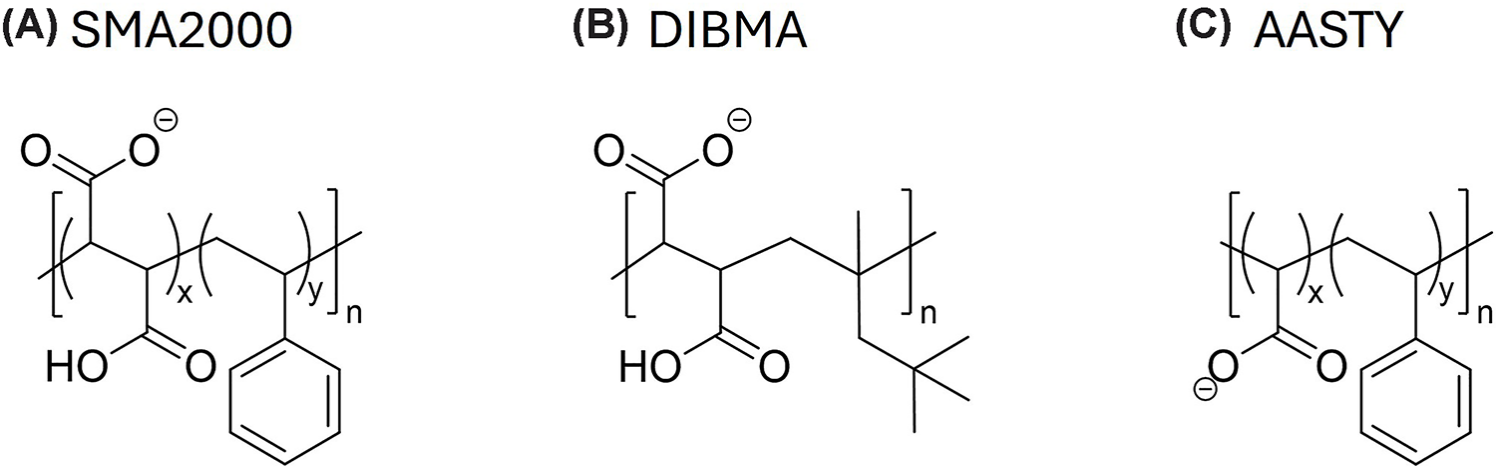
Chemical structures of the polymers (**A**) SMA2000, which has a 2:1 ratio of styrene to maleic acid. (**B**) DIBMA, which has a diisobutylene group instead of the styrene, and a 1:1 ratio of diisobutylene to maleic acid. (**C**) AASTY, which has acrylic acid in place of maleic acid, and a ratio of styrene:acrylic acid ranging from 1.2:1 to 0.8:1 depending on the specific polymer.

This study utilised a range of commercially available AASTY polymers. These are named according to their approximate molecular weight (6 or 11 kDa), and the acrylic acid proportion of the polymer (45%, 50%, or 55% acrylic acid). They were compared with commercially available DIBMA, and the original SMA2000 (Figure 1 and Supplementary Table S1).

**Figure 2:**
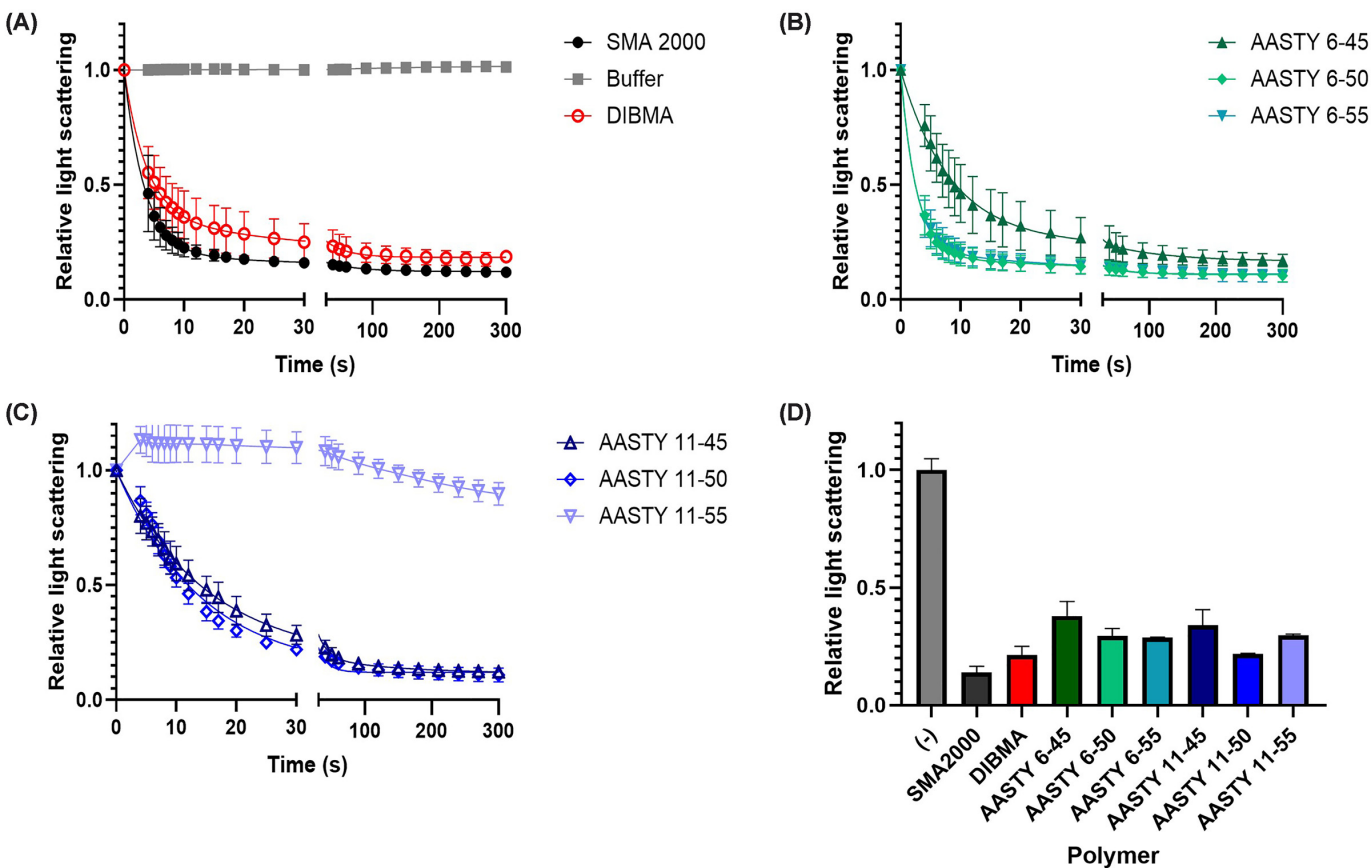
AASTY polymers effectively solubilise Sf9 insect cell membranes but with different kinetics Solubilisation of Sf9 insect cell membranes (30 mg/ml wet pellet weight) with each polymer (2.5% (w/v) for SMA2000 and DIBMA, 0.5% (w/v) for AASTYs) was monitored via light scattering over time at a wavelength of 390 nm at room temperature. (**A**) SMA2000 and DIBMA compared to a buffer only control. (**B**) The 6kDa AASTY polymers. (**C**) The 11 kDa AASTY polymers. (**D**) The final light scattering for each sample after a 1 h incubation at room temperature. Data are mean ± SD, *n* ≥ 3.

We first investigated how effective the various polymers were at solubilising biological membranes using a light scattering assay. Membrane vesicles derived from Sf9 cells scatter light at 390 nm, but PLPs that are much smaller do not. Figure 2A–C shows the kinetics of solubilisation of Sf9 insect cell membranes, measured by light scattering, at room temperature. SMA2000 and DIBMA were used at the standard final concentration of 2.5% (w/v), whereas the AASTY polymers were used at a final concentration of 0.5% (w/v) as recommended by the suppliers. It can be seen in Figure 2A that SMA2000 solubilised the membranes very rapidly, reaching a plateau after approximately 10 s, and DIBMA was marginally slower. AASTY 6-50 and AASTY 6-55 solubilised the membranes comparably to SMA2000, whilst AASTY 6-45 was a little slower (Figure 2B). AASTY 11-45 and AASTY 11-50 solubilised the membranes slower than SMA2000 but still achieved substantial solubilisation within 30 s. In contrast, AASTY 11-55 showed an initial increase in light scattering before slowly decreasing over time (Figure 2C). However, after a 1 h incubation, all of the polymers tested showed a substantial and comparable decrease in light scattering (Figure 2D).

### Solubilisation of specific membrane proteins and lipids using AASTY polymers

**Figure 3:**
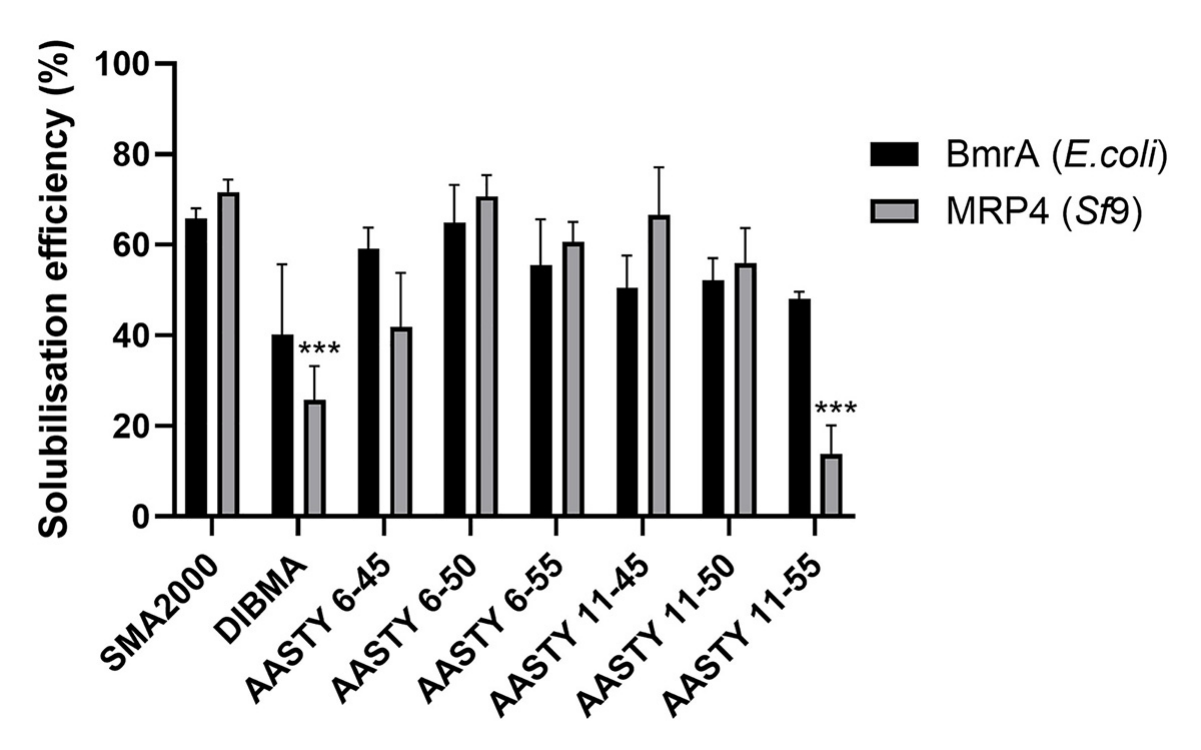
AASTY polymers can extract ABC transporters from *E. coli* and Sf9 insect cells BmrA and MRP4 were recombinantly expressed in C41 *E. coli* and Sf9 insect cells, respectively. Membranes from each were mixed with polymer for 1 h at room temperature, and then ultracentrifuged. Samples of the supernatant (solubilised protein) and resuspended pellet (insoluble material) were analysed by western blot using an anti-his antibody for BmrA or an anti-MRP4 antibody. Blots were analysed by densitometry to calculate the solubilisation efficiency. Data are mean ± SEM, *n* ≥ 3. Data were analysed using a two-way ANOVA with a Tukey posthoc test. ***P <0.001 significantly different to SMA2000.

The next step was to analyse the solubilisation of specific ABC transporter proteins from different expression systems. BmrA was expressed in *E. coli* and MRP4 was expressed in Sf9 insect cells. Figure 3 shows that most of the AASTY polymers performed comparably to SMA2000 for solubilisation of both proteins. The one exception was AASTY 11-55, which showed a statistically significant lower extraction of MRP4. DIBMA appeared to give a lower solubilisation efficiency for both proteins, although it was only significant for MRP4, this is comparable to previous studies using DIBMA [19]. To complement the solubilisation of specific proteins, we also tested whether the polymers showed any preferences in terms of lipid extraction from either *E. coli* or Sf9 cells using TLC. Whilst the profile of lipids present in Sf9 cells is quite different to that in *E. coli*, no differences were observed in the relative ratio of the main lipids in the total membrane samples compared to those extracted with the polymers (Supplementary Figure S1). For *E. coli*, the predominant lipid species were PE and PG, and the ratio of PE:PG was 2.2–2.8, which agrees well with commercial lipid extracts (https://avantiresearch.com/product/100600). For Sf9 cells, the ratio of PE:PC was 1.6–2.2, which agrees well with previous studies [25]. These data suggest that the polymers show no preferential extraction of specific lipids.

### BmrA affinity purification yield and purity

**Figure 4:**
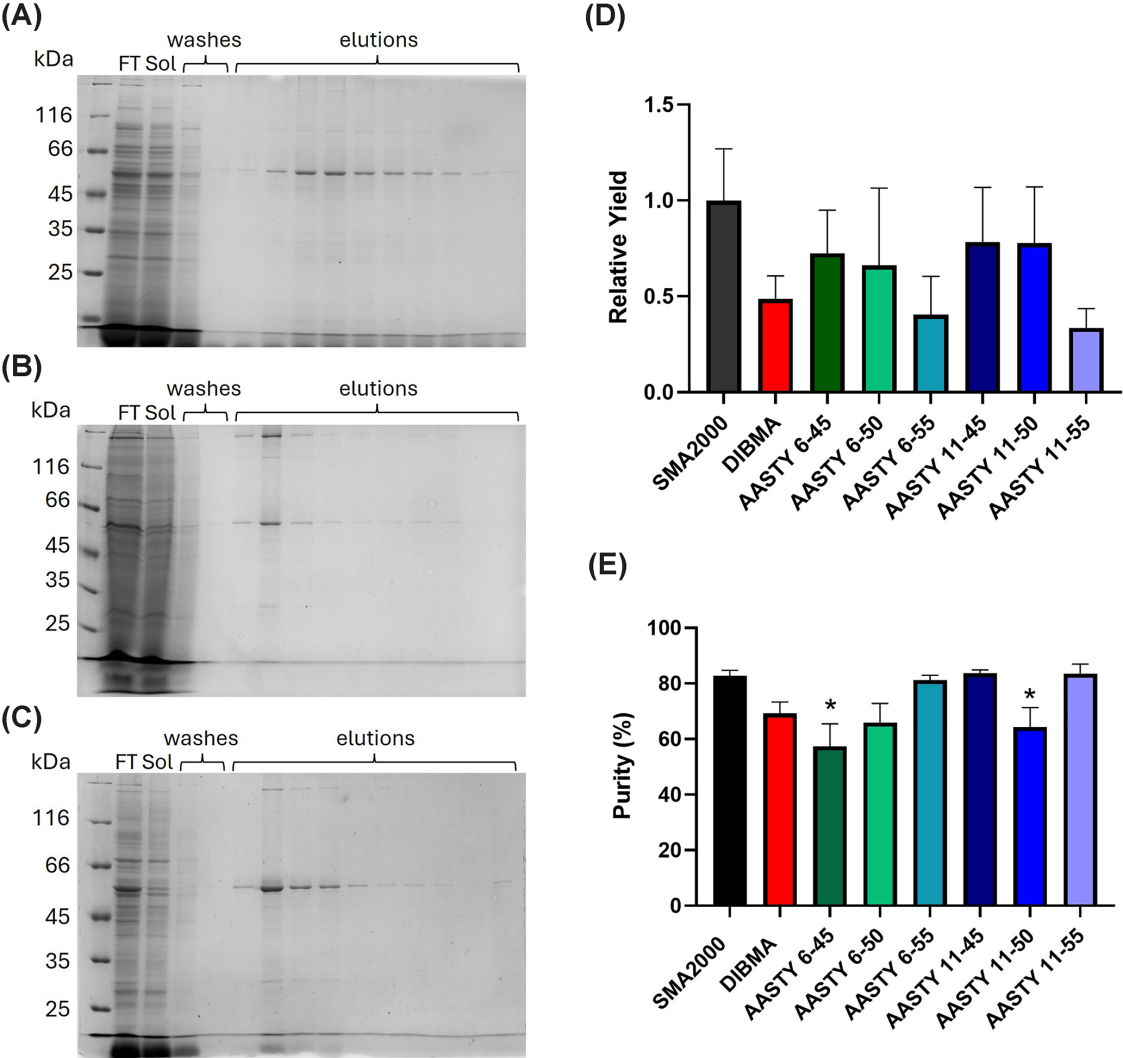
BmrA was successfully purified with AASTY polymers BmrA expressing *E. coli* membranes were solubilised with each polymer, solubilised proteins were mixed with Ni-NTA resin overnight at 4°C, transferred to a gravity column, and washed with 40 bv buffer containing 20 mM imidazole. Proteins were eluted in 0.5 bv fractions using buffer containing 150 mM imidazole. Samples were run on SDS–PAGE and stained using Instant Blue. (**A–C**) Representative purification gels using SMA2000 (**A**), DIBMA (**B**), or AASTY 6-50 (**C**). (**D**) The yield of purified protein was quantified by densitometric analysis of samples run on SDS–PAGE alongside BSA standards (0.25–1.5 μg). All values obtained were corrected for membrane batch-to-batch variation in yield by normalising to the values obtained with SMA2000 using the same membrane preparation. (**E**) The degree of purity achieved following affinity purification was measured by densitometric analysis (ImageJ) of a single lane of purified protein run on SDS–PAGE. Data are mean ± sem, *n* ≥ 3. Data were analysed using a one-way ANOVA with a Tukey posthoc test, *P < 0.05 significantly different to SMA2000.

We then moved on to examine the use of the polymers for purifying BmrA using affinity chromatography. Example SDS–PAGE of the purification steps are shown in Figure 4A–C. The elution fractions with SMA2000 contain a relatively clean band corresponding to BmrA (Figure 4A). With DIBMA BmrA is also purified, but the yield appears a lot less (Figure 4B). For AASTY 6-50 (Figure 4C), the yield appears to be higher than for DIBMA. Interestingly for both DIBMA and AASTY 6-50, the protein eluted from the column in earlier elution fractions than with SMA2000. When looking at average yield data (Figure 4D), although DIBMA, AASTY 6-55, and AASTY 11-55 appear to give lower yields, the variation for all conditions is large and no differences are statistically significant.

### Characterization of AASTY PLPs

**Figure 5:**
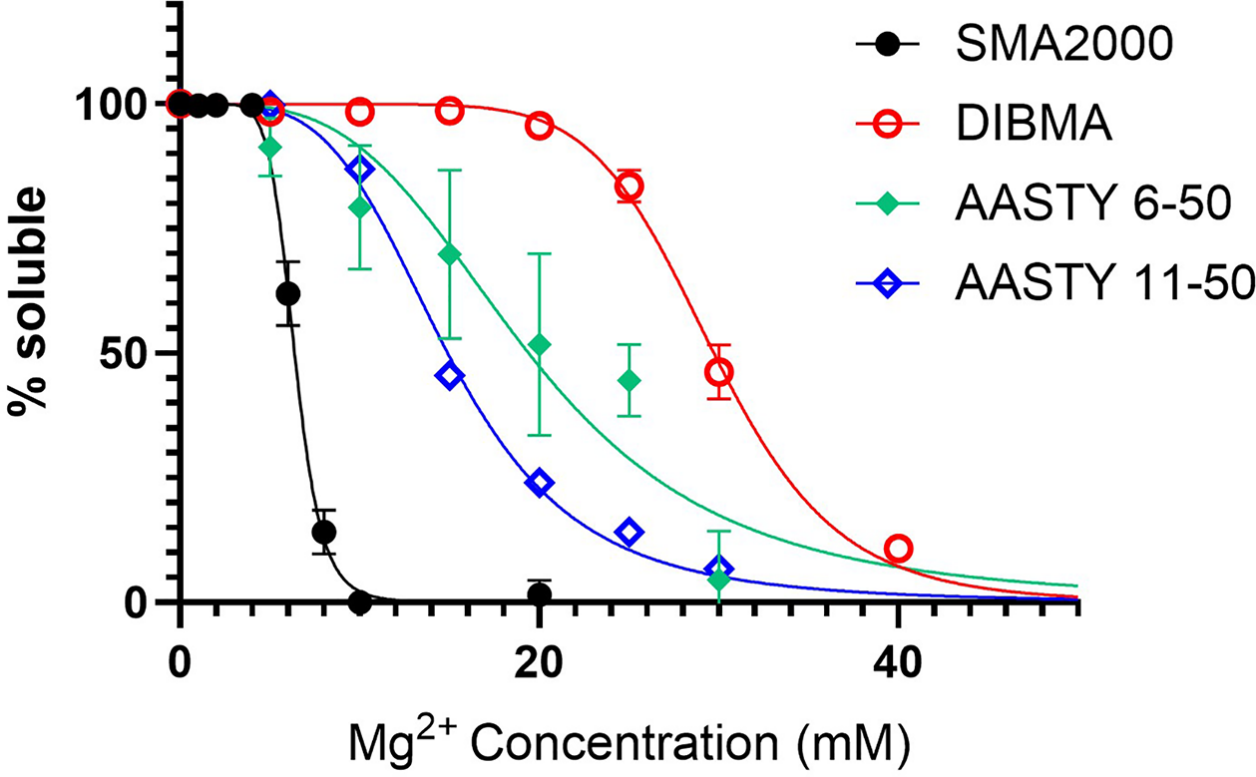
AASTY and DIBMA polymers have an increased tolerance to Mg^2+^ compared to SMA2000 Lipid only PLPs were formed from DMPC with each polymer and purified by SEC. They were mixed with MgCl_2_ (0–10 mM) in a 96-well plate and light scattering measured at 395 nm. Data are mean ± SEM (*n* ≥ 3), and data were fitted with a normalised dose-response curve.

It was previously reported that the AASTY polymers offered an increased tolerance to divalent cations [16,17], as does DIBMA [18,19]. Figure 5 shows that we also observed this increased tolerance. SMA2000 PLPs were 50% precipitated at 6 mM Mg^2+^, whilst for AASTY 6-50, AASTY 11-50, and DIBMA PLPs, this had shifted to 19 mM, 15 mM, and 30 mM, respectively. Similar curves for all of the AASTY variants are shown in Supplementary Figure S2. Most behaved comparably, but notably AASTY 6-45 did not show as strong a tolerance to Mg^2+^ as the other polymers.

Taking the results of the solubilisation, purification yield and Mg^2+^ tolerance assays together it was decided to proceed with just AASTY 6-50 and AASTY 11-50 for further characterisation of the discs and investigation of ABC transporter function.

**Figure 6:**
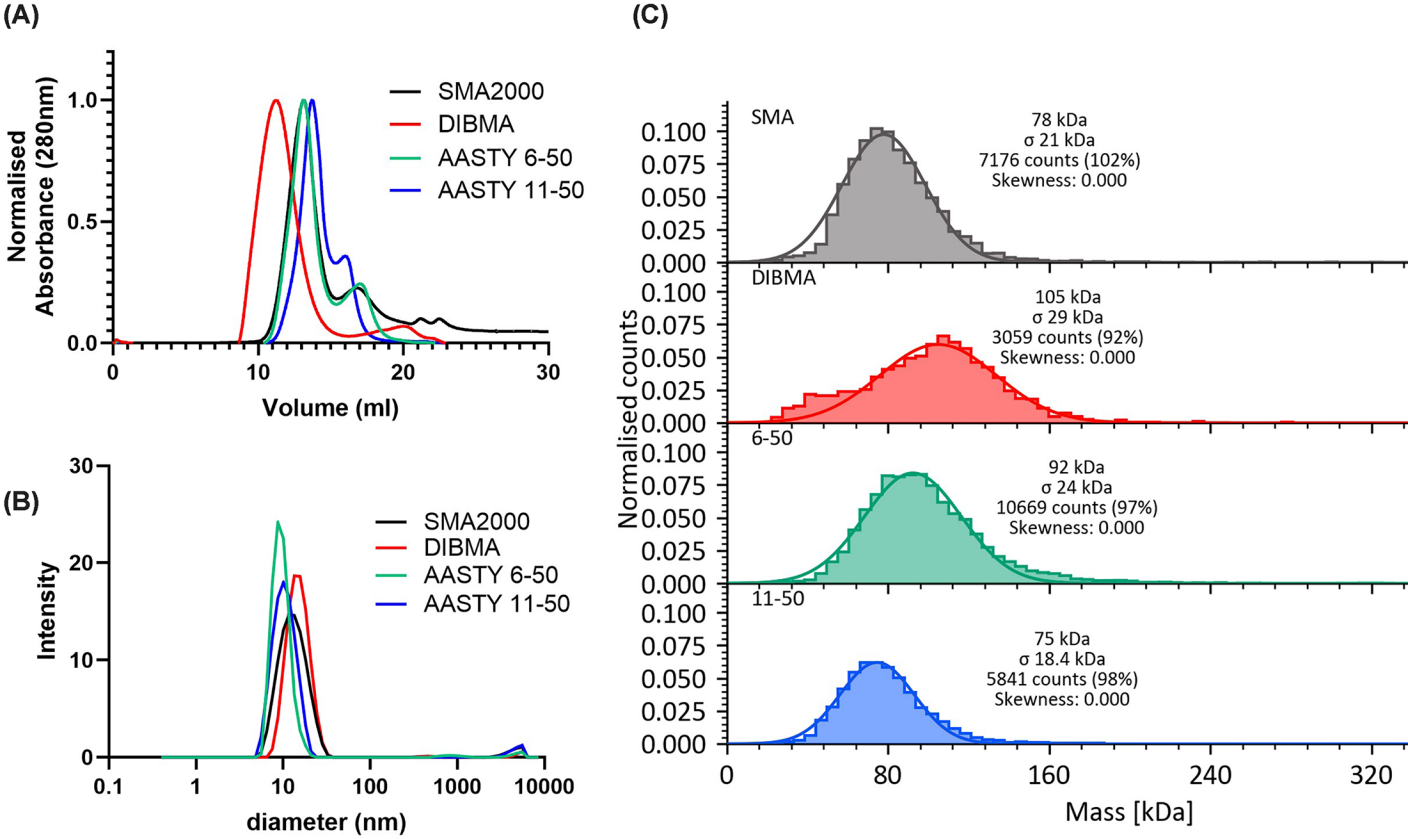
AASTY PLPs are comparable in size to SMALPs, but DIBMALPs are larger Lipid only PLPs were formed with each polymer and analysed by SEC (**A**), DLS (**B**), and mass photometry (**C**).

In Figure 6, three different methods were used to examine the size of the PLPs formed by each polymer with DMPC lipids. Firstly, size exclusion chromatography shows that the peaks for AASTY 6-50 and SMA2000 PLPs were overlapping at approximately 12 ml, whilst AASTY 11-50 PLPs eluted slightly later. DIBMALPs eluted earlier at approximately 10 ml implying larger discs, as has been previously shown [18,19]. DLS showed that the average hydrodynamic diameter of PLPs formed with the AASTY polymers was approx. 10 nm, as has been shown previously for SMALPs [10,19]. It also showed that DIBMALPs were the largest (15.3 nm), although the difference was less than seen on the SEC. Finally, mass photometry analyses showed that SMA2000 and AASTY 11-50 PLPs gave comparable size distributions, with AASTY 6-50 being slightly larger. DIBMALPs showed a wider distribution than the other polymers and again showed that they were the largest particles. Taking the results of all three methods together, it was concluded that the two AASTY polymers form discs of comparable size to SMA2000, whilst DIBMA forms larger discs.

**Figure 7:**
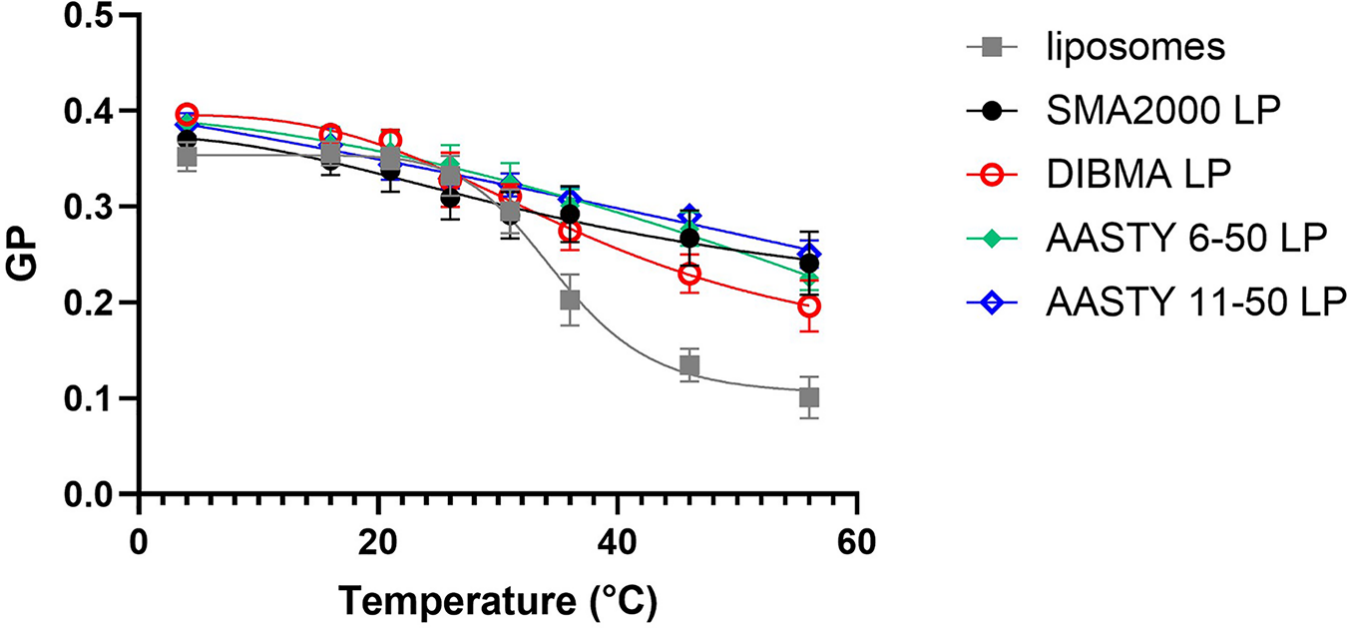
Lipids within PLPs are more rigid than within liposomes DMPC liposomes (0.1% w/v) were mixed with 10 μM of laurdan. Following incubation at various temperatures, the fluorescence spectra was measured (ex. 350 nm, em. 380–600 nm). The fluorescent intensities at 435 and 490 nm were used to calculate the GP value. For PLPs, 0.2% (w/v) polymer was added to the DMPC/laurdan mix for 30 min. Data are mean ± SEM, *n* ≥4.

The next feature we wanted to characterise was the fluidity of lipids within the discs. To assess this, the laurdan assay was used. The laurdan fluorophore intercalates within the lipid bilayer and its fluorescence spectrum changes dependent on the degree of polarity around the fluorescent group. From these changes, the GP can be calculated, which can be used as proxy for membrane fluidity as fluid membranes have higher concentrations of water near the lipid headgroups and the laurdan fluorescent moiety [26]. As shown in Figure 7, the GP for DMPC in liposomes is 0.35 at 4°C, showing a relatively rigid membrane. As the temperature increases, the GP decreases with a characteristic sigmoidal curve, as the lipids pass the phase transition to a more fluid state. DMPC within SMALPs give a comparable GP to liposomes at 4°C, but as the temperature increases the GP does not decrease as much, and there is no sigmoidal phase transition, suggesting lipids within the SMALPs are in a more rigid environment than in liposomes. The lipid particles formed using the AASTY polymers behaved comparably to those in SMA2000. For DIBMALPs, the curve is similar to SMA, but there is more of a sigmoidal shape to the curve, and the GP at the higher temperatures is lower than with the other PLPs, but still significantly higher than in liposomes. This suggests that DIBMALPs may allow a slightly more fluid lipid environment than the other polymers, but that it is still more rigid than in liposomes.

### Functional analysis of BmrA in AASTY PLPs

**Figure 8:**
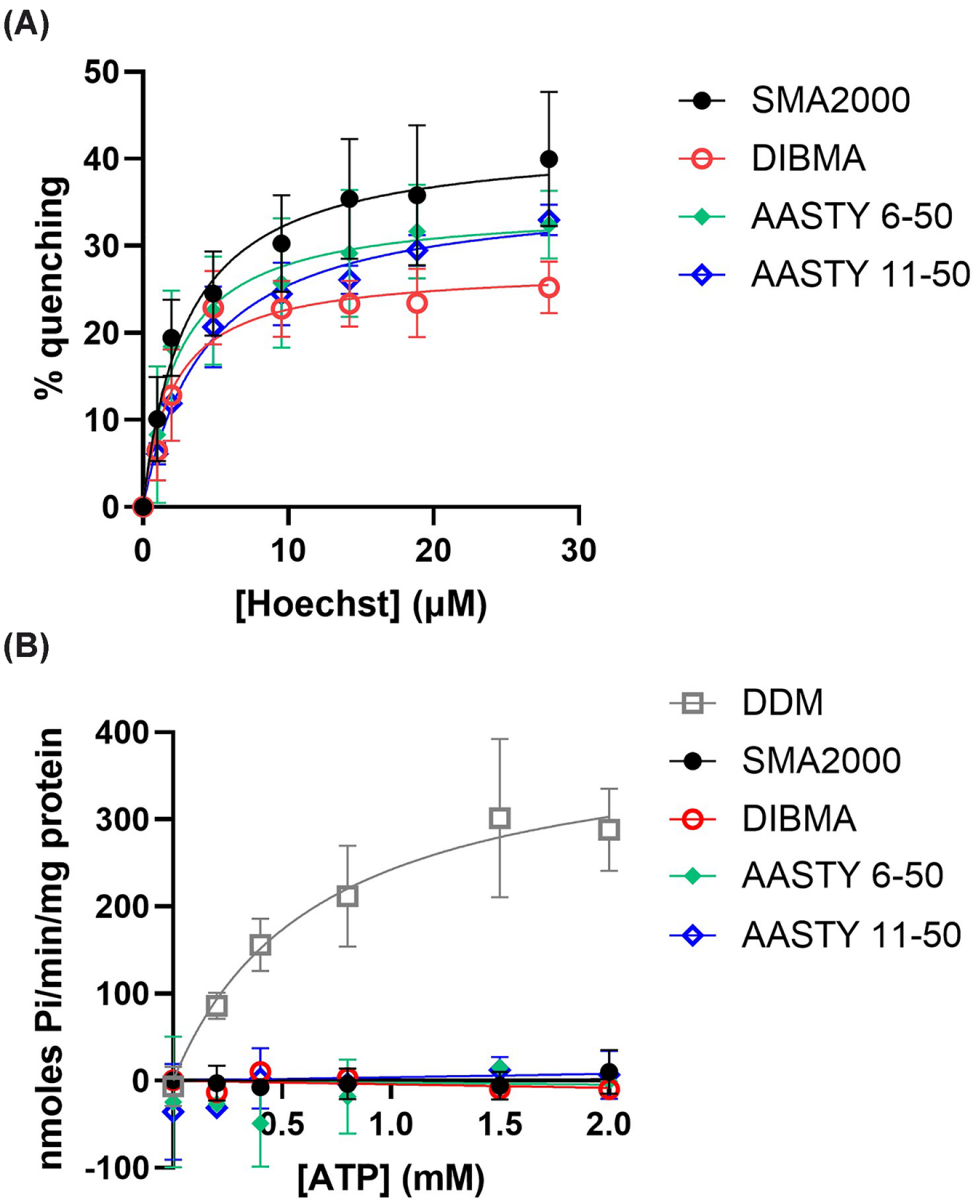
BmrA within AASTY or DIBMA PLPs binds ligands but do not show ATPase activity (**A**) Binding of the substrate Hoechst 33342 to purified BmrA was analysed by tryptophan fluorescence quenching. Data are mean ± SEM, *n* ≥ 3. (**B**) ATPase activity of BmrA purified within PLPs or DDM micelles was assayed by a colourimetric Pi release assay. Data are mean ± SEM, *n* ≥ 3.

Finally, we wanted to investigate whether ABC transporters within AASTY PLPs retained function. Ligand binding (Hoecsht 33342) was monitored using a tryptophan fluorescence quenching assay (Figure 8A). BmrA within SMALPs had a *K*_D_ of 2.6 ± 0.8 μM and a maximal quenching of 46 ± 9%, which are comparable with previous studies [10]. BmrA within the AASTY and DIBMA PLPs showed similar binding curves to SMA2000, with no statistically significant differences between any of them for *K*_D_ or maximal quenching. This suggests that BmrA in each PLP binds ligand comparably.

Whilst ligand binding shows that the protein is likely folded in the correct way and retains the ability to bind substrates effectively, it is a rather static measurement. ABC transporters are very dynamic proteins that undergo fairly extensive conformational changes during their function, which are powered by the binding and hydrolysis of ATP. Therefore, ATPase activity provides a more robust measure of overall ABC protein function. Within conventional SMALPs, this has been challenging to achieve due to the Mg^2+^ sensitivity. Mg^2+^ is needed as a cofactor for the ATPase activity, yet it makes SMALPs fall apart and then protein precipitates from solution [12]. However, the AASTY and DIBMA polymers showed enhanced tolerance to Mg^2+^, so we tested whether these PLPs were amenable to measuring BmrA ATPase activity. As a positive control for the ATPase assay BmrA was purified in DDM micelles, as it is known that ATPase activity can be measured in DDM [6]. Details of solubilisation and purification of BmrA using DDM compared to SMA2000 can be found in Supplementary Figure S3, which is comparable to previous reports [10]. Figure 8B shows that BmrA within DDM micelles shows a typical Michaelis–Menten curve with a Km for ATP of 0.66 ± 0.03 mM and a Vmax of 403 ± 51 nmoles Pi/min/mg protein, which is comparable to previous reports [6,27–29]. However, BmrA within all of the PLPs showed no ATPase activity at all.

## Discussion

The aim of this study was to test commercially available AASTY and DIBMA polymers for extraction, purification, and functional analysis of ABC transporters. All polymers tested were able to solubilise biological membranes effectively. However, the differences seen in the kinetics of solubilisation of Sf9 insect cell membranes are interesting. It has previously been shown for SMA-based polymers that lower molecular weight polymers solubilise membranes faster than larger molecular weight polymers [13,30,31] and this was also observed here, with the 11 kDa AASTY polymers showing slower kinetics than the 6 kDa AASTY polymers (Figure 2). Perhaps, more surprising was the big difference observed with AASTY 11-55 polymer compared to the other AASTY polymers. The differences between 45%, 50%, and 55% acrylic acid content of the polymers seems relatively small, and for the 6 kDa polymers there are only subtle differences in the kinetics of solubilisation. However, for the 11 kDa polymers the most polar 55% acrylic acid polymer solubilised Sf9 membranes much more slowly than the other 11 kDa AASTY polymers. In fact, an initial increase in light scattering was observed. This has previously been observed with the larger benzylamine-modified SMA polymers, and was hypothesised to reflect the initial association of the polymer with liposomes or the initial steps of solubilisation [13]. Although it is not possible to observe this initial increase with polymers that show faster kinetics, it is possible that it is still happening. It is known that the ratio of hydrophobic to hydrophilic components within polymers, and the length of the polymers are both critical factors for whether a polymer works for solubilising membranes [10]. It seems that the combination of the 11 kDa larger size, along with the 55% polar acrylic acid component is less ideal for solubilising insect cell membranes than the other polymer structures. This is also reflected in the significantly lower solubilisation efficiency for MRP4 expressed in insect cells (Figure 3). However, the AASTY 11-55 works more efficiently for extracting BmrA from *E. coli* membranes where it shows no statistical difference to the other AASTY polymers or SMA2000. *E. coli* membranes contain quite distinct lipids from Sf9 cells, so perhaps these differences simply reflect a combination of polymer properties and the lipid properties of the membrane being solubilised. The effectiveness of DIBMA was comparable to previously reported results, where it showed a lower solubilisation efficiency and purification yield for some proteins [19]. However, it should be noted that the commercially available DIBMA polymer was much easier to use and more consistent than preparing it from Sokalan CP9.

It was previously shown that SMA2000 works very well for extracting and purifying ABC transporters, showing a greater degree of protein purity than detergent, and a greater protein stability [12]. However, it was not possible to measure function in terms of ATPase activity due to the Mg^2+^ sensitivity of SMA2000. Both AASTY and DIBMA polymers have previously been shown to have an increased tolerance to Mg^2+^ compared to SMA2000 [16–19]. So, it was hypothesised that they may enable assessment of ATPase activity of purified ABC transporters. In this study, we confirmed the increased tolerance to Mg^2+^ for both AASTY and DIBMA polymers (Figure 5). Despite this, we were still not able to measure any ATPase activity for BmrA purified in either AASTY or DIBMA PLPs (Figure 8). This was not an issue with the expression conditions or the protein itself, since BmrA from the same membrane preparations purified in DDM showed ATPase activity comparable with previous reports [6, 27, 28, 29]. Therefore, it is not simply the divalent cation sensitivity of polymers that prevents measurement of ATPase activity.

This leads to the question of what is the reason underlying why ABC transporters within PLPs do not display ATPase activity? One possible explanation could be that the protein is too restricted within the PLPs to undergo the relatively large-scale conformational changes that accompany ATP binding and hydrolysis [32,33]. It is known that the properties of the lipid environment, including bilayer thickness, headgroups, and membrane curvature, impact the ATPase activity of BmrA [28]. SMALPs confer greater stability to encapsulated proteins, but this could be at the cost of restricting dynamics. It was previously shown that ABC transporters within membrane scaffold protein (MSP) nanodiscs can display ATPase activity, but that the activity is affected by the nanodisc size, with larger nanodiscs being preferred [34], and that the conformation of an ABC transporter within MSP nanodiscs was more restricted than in detergent micelles [35]. Similarly, rhodopsin within SMALPs could not undergo its full photoactivation pathway and became stuck in an intermediate state; however, rhodopsin within DIBMALPs could undergo its full activation [15]. DIBMALPs are known to be larger than SMALPs, as we also saw in the present study (Figure 6), and it has previously been reported that the lipids within DIBMALPs are more fluid than in SMALPs [18], which could explain why DIBMALPs allowed full conformational changes for rhodopsin whereas SMALPs did not. In our study, the laurdan assay showed that lipids within DIBMALPs showed hints of the phase transition expected for DMPC, which was not at all apparent for lipids in SMALPs or AASTY PLPs (Figure 7). However, it was still significantly different to the lipids within liposomes. It should be noted that both our laurdan assay and the previous study examined DIBMALPs with lipids only encapsulated. Inclusion of an ABC transporter within the disc will mean fewer lipids are present, and the lipids around the edge of the disc which interact with the polymer are known to be more rigid than the lipids in the centre [36]. Furthermore, BmrA is larger (12 transmembrane helices) than rhodopsin (7 transmembrane helices) and the conformational changes it undergoes are larger. Taken together this may explain why DIBMA works for full function of rhodopsin but not for BmrA. The AASTY PLPs were comparable in size and lipid fluidity to SMALPs, so they likely offer the same limitations.

An alternative suggestion for the lack of ATPase activity could simply be the high negative charge of the polymers provided by the maleic acid and acrylic acid groups. When the PLPs form and the polymer wraps around the lipid disc, the acid groups are on the outside of the disc and confer solubility to the disc in solution. This high density of negative charges may cause issues with binding of ATP which is also negatively charged. It has previously been shown that the polyanionic nature of both SMALPs and DIBMALPs leads to non-specific interactions, and they interfere with charge-sensitive protein–lipid interactions [37]. Although it has previously been reported that the related protein P-glycoprotein (ABCB1) within SMALPs was able to bind ATP using a tryptophan fluorescence quenching assay [12]. Even so, in future it may be interesting to test polymer variants that do not have this high negative charge.

In conclusion, the AASTY polymers work comparably to SMA2000 for the extraction and purification of ABC transporters. They offer an increased tolerance to divalent cations, but this is not sufficient to be able to measure ATPase activity of encapsulated ABC transporters.

## Supplementary Material

Supplementary Table S1 and Figures S1-S3

## Data Availability

The underlying data for this study can be found at the Aston Explorer Data Repository DOI: 10.17036/researchdata.aston.ac.uk.00000662.

## Abbreviations

AASTY: Acrylic acid styrene co-polymer
ABC transporter: ATP-binding cassette superfamily of transporters
ABCC4: ABC protein subfamily C member 4
ATPase: adenosine triphosphate hydrolysis activity
BmrA: multidrug efflux transporter of ABC superfamily from *Bacillus subtilis*
BSA: bovine serum albumin
BV: bed volume
CL: cardiolipin
DIBMA: diisobutylene maleic acid co-polymer
DIBMALP: DIBMA lipid particle
DLS: dynamic light scattering
DMPC: 1,2-dimyristoyl-*sn*-glycero-3-phosphocholine
*E. coli*: *Escherichia coli*
LB-amp: Luria broth supplemented with 100 μg/ml of ampicillin
M_n_: number-average molecular weight
MRP4: multidrug resistance protein 4
MTBE: methyl-tert-butyl ether
MSP: membrane scaffold protein
Ni-NTA: nickel nitriloacetic acid
PC: phosphatidylcholine
PE: phosphatidylethanolamine
PG: phosphatidylglycerol
Pi: inorganic phosphate
PLP: polymer lipid particle
PS: phosphatidylserine
SDS–PAGE: sodium dodecyl sulphate–polyacrylamide gel electrophoresis
SEC: size exclusion chromatography
Sf9: *Spodoptera frugiperda* cell line
SMA: styrene maleic acid co-polymer
SMALP: SMA lipid particle
SMAnh: styrene maleic anhydride
TLC: thin-layer chromatography
