# Supplementary material for: Overcoming divalent cation sensitivity is not the only challenge for functional study of ABC transporters within polymer lipid particles
Source: Biosci Rep. 2026 Feb 4;46(2):BSR20250256. doi: 10.1042/BSR20250256 (PMC13078315; doi:10.1042/BSR20250256)
Supplement: Supplementary Table S1 and Figures S1-S3 [file BSR-2025-0256_supp.pdf]

Aston University
BIRMINGHAM UK

College of Health and Life Sciences

Aston University
Birmingham B4 7ET
United Kingdom

+44 (0)121 204 3000
aston.ac.uk

9th January 2026

We would like to submit the enclosed revised manuscript: **'Overcoming divalent cation sensitivity is not the only challenge for functional study of ABC transporters within polymer lipid particles** for publication in Bioscience Reports.

We thank the reviewers for taking the time to evaluate our manuscript and appreciate the feedback they have provided. We have revised the manuscript accordingly and included a point by point response to their comments.

Please contact me if you have any questions or require further information.

Yours sincerely,

Dr. Alice Rothnie
Reader in Biochemistry
Tel: +44 (0)121 204 4013
a.rothnie@aston.ac.uk

Supplementary Information

Overcoming divalent cation sensitivity is not the only challenge for functional study of ABC transporters within polymer lipid particles.

Olivia P Hawkins, Thomas J Potter, Luke M Broadbent, Philip Kitchen, Alan D Goddard and Alice J Rothnie*

Aston Institute for Membrane Excellence & Department of Biosciences, Aston University, Birmingham, UK.

* corresponding author: Dr Alice Rothnie (a.rothnie@aston.ac.uk)

Supplementary Table 1. Properties of the polymers used in this study. The ratio of the different components of each polymer, and number averaged molecular weight (Mn) as provided by the suppliers. [a]Mn was determined experimentally by Oluwole et al. (2017) Angew. Chem. Int. Ed. 56, 1919-1924.

**Name**	**Supplier**	**Non-polar**	**Polar**	**Ratio non-polar:polar**	**Mn (kDa)**
SMA2000	Cray Valley	Styrene	Maleic acid	2:1	3
DIBMA	Glycon	Diisobutylene	Maleic acid	1:1	8.4[a]
AASTY 6-45	Cube Biotech	Styrene	Acrylic acid	1.2:1	5.5
AASTY 6-50	Cube Biotech	Styrene	Acrylic acid	1:1	5.5
AASTY 6-55	Cube Biotech	Styrene	Acrylic acid	0.8:1	5.5
AASTY 11-45	Cube Biotech	Styrene	Acrylic acid	1.2:1	10-11
AASTY 11-50	Cube Biotech	Styrene	Acrylic acid	1:1	10-11
AASTY 11-55	Cube Biotech	Styrene	Acrylic acid	0.8:1	10-11

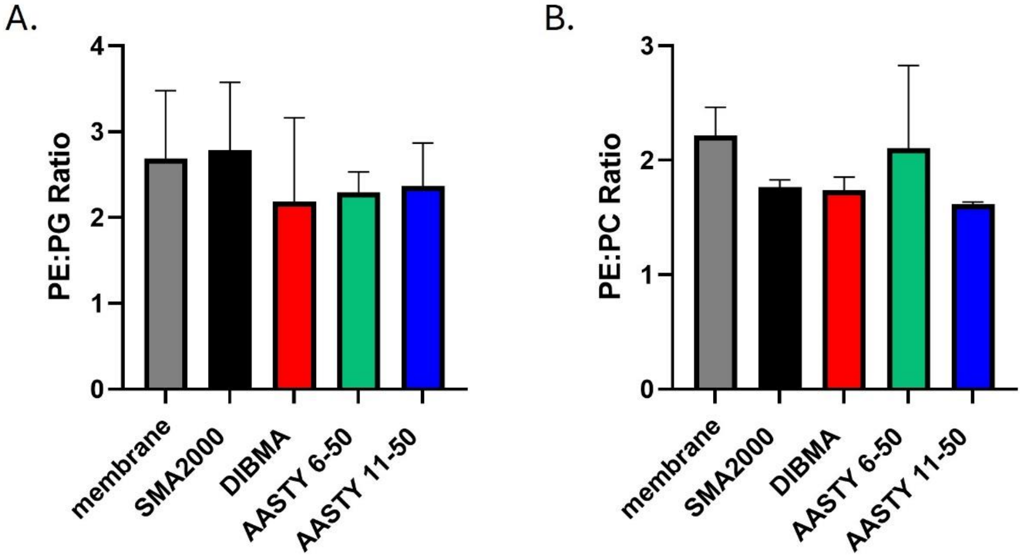

Supplementary Figure 1. The polymers do not show preferential lipid extraction. Membrane preparations from *E.coli* cells (A) or Sf9 insect cells (B) were mixed with each polymer, and the soluble fraction harvested by ultracentrifugation. The lipids in the solubilised fraction were solvent extracted using MTBE and methanol, and analysed by TLC against purified lipid standards. The TLC plate was stained with molybdenum blue and imaged. The proportion of PE:PG (A) or PE:PC (B) were determined by densitometry. Data are mean ± SD, n≥2.

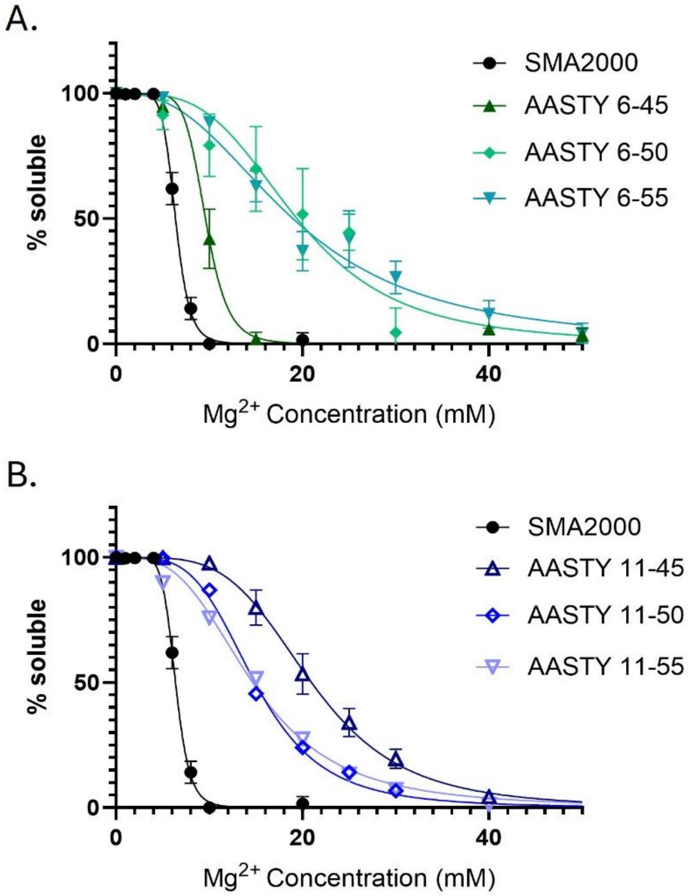

Supplementary Figure 2. AASTY polymers have an increased tolerance of Mg^{2+} compared to SMA2000. Lipid only PLPs were formed from DMPC with each polymer and purified by SEC. They were mixed with $MgCl_2$ (0-10 mM) in a 96-well plate and light scattering measured at 395 nm. Data are mean ± SEM (n≥3), and data were fitted with a dose-response curve.

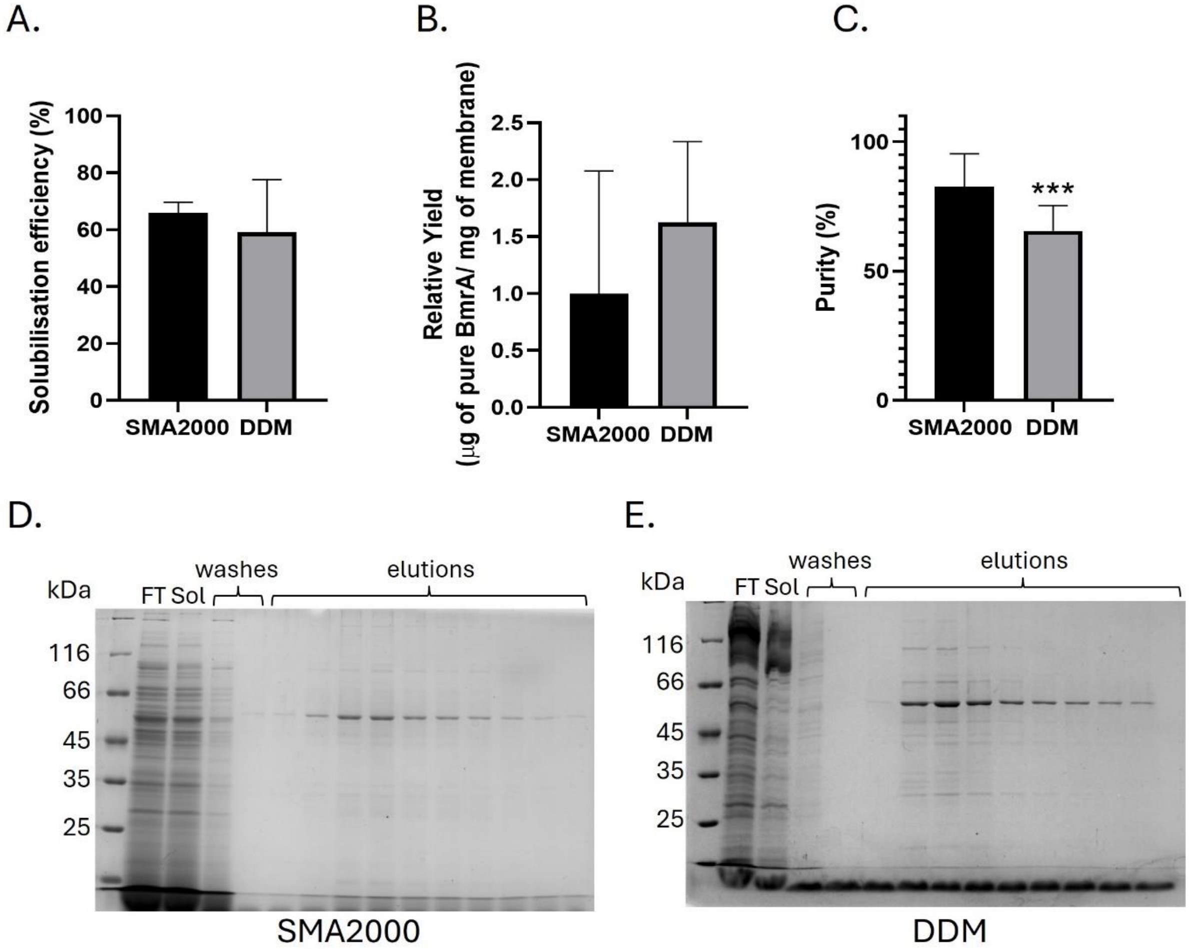

Supplementary Figure 3. Comparison of SMA2000 and DDM for the solubilisation and purification of BmrA. A; Membranes from *E.coli* overexpressing BmrA were solubilised with 2.5% SMA2000 or 2% DDM for 1 hour. Following ultracentrifugation samples of the supernatant containing soluble protein and the pellet containing insoluble material were run on Westerns blots, probed with an anti-his antibody and analysed by densitometry. Data are mean±SD, n=3. B-E; BmrA solubilised using either SMA2000 or DDM were purified by Ni-affinity chromatography. B; Yield of purified protein was determined from densitometric analysis of SDS-PAGE using BSA standards. Data are mean±SD, n≥4. C; Purity of purified BmrA was measured by densitometric analysis of elution lanes on SDS-PAGE. Data are mean±SD, n≥11. D; Representative SDS-PAGE showing the purification of BmrA when solubilised with SMA2000. E; Representative SDS-PAGE showing the purification of BmrA when solubilised with DDM. Data were analysed by t-test, ***p<0.01.
